# A novel mindful-compassion art therapy (MCAT) for reducing burnout and promoting resilience for end-of-life care professionals: a waitlist RCT protocol

**DOI:** 10.1186/s13063-019-3533-y

**Published:** 2019-07-08

**Authors:** Andy Hau Yan Ho, Geraldine Tan-Ho, Thuy Anh Ngo, Grace Ong, Poh Heng Chong, Dennis Dignadice, Jordan Potash

**Affiliations:** 1 Psychology Programme, School of Social Sciences, Nanyang Technological University, 14 Nanyang Drive, HSS-04-03, Singapore, 637332 Singapore; 2 Centre for Population Health Sciences, Lee Kong Chian School of Medicine, 11 Mandalay Road, Level 18, Clinical Science Building, Singapore, 308232 Singapore; 3 The Palliative Care Centre for Excellence in Research and Education, 10 Jalan Tan Tock Seng, Singapore, 308436 Singapore; 4 Assisi Hospice, 832 Thomson Road, Singapore, 574627 Singapore; 5 HCA Hospice Care, 12 Jalan Tan Tock Seng, Singapore, 308437 Singapore; 6 Art Therapy Programme, Columbian College of Arts and Sciences, George Washington University, 413 John Carlyle Street, Second Floor, Alexandria, VA 22314 USA

**Keywords:** Burnout, Resilience, Mindful compassion, Art therapy, Multimodal intervention, End-of-life care, Palliative care, Randomized control trial

## Abstract

**Introduction:**

End-of-life (EoL) care professionals are prone to burnout given the intense emotional nature of their work. Previous research supports the efficacy of art therapy in reducing work-related stress and enhancing emotional health among professional EoL caregivers. Integrating mindfulness meditation with art therapy and reflective awareness complementing emotional expression has immense potential for self-care and collegial support. Mindful-compassion art therapy (MCAT) is a novel, empirically informed, and highly structured intervention that aims to reduce work-related stress, cultivate resilience, and promote wellness. This study aims to assess the potential effectiveness of MCAT for supporting EoL care professionals in Singapore.

**Methods:**

This is an open-label waitlist randomized controlled trial. Sixty EoL care professionals, including doctors, nurses, social workers, and personal care workers, are randomly allocated to one of two groups: (i) an intervention group that receives MCAT immediately and (ii) a waitlist-control group that receives MCAT after the intervention group completes treatment. Face-to-face self-administered outcome assessments are collected at three different time points—baseline (T1) for both groups, post-intervention (T2), and 6-week follow-up (T3) for intervention group—as well as pre-intervention (T2) and post-intervention (T3) for the waitlist-control group. The primary outcome measure is burnout, and secondary measures include emotional regulation, resilience, compassion, quality of life, and death attitudes. Between- and within-participant comparisons of outcomes are conducted, and the appropriate effect size estimates are reported. An acceptability and feasibility study is to be conducted by using a triangulation of qualitative data with framework analysis.

**Discussion:**

The outcomes of this study will contribute to advancements in both theories and practices for supporting professional EoL caregivers around the world. It will also inform policy makers about the feasibility, acceptability, and effectiveness of delivering a multimodal psycho-socio-spiritual intervention within a community institutional setting. The study has received ethical approval from the institutional review board of Nanyang Technological University.

**Trial registration:**

ClinicalTrials.gov Identifier: NCT03440606. Retrospectively registered February 21, 2018.

**Electronic supplementary material:**

The online version of this article (10.1186/s13063-019-3533-y) contains supplementary material, which is available to authorized users.

## Background

Professional caregivers working in the fields of end-of-life (EoL), palliative, and bereavement care are regularly exposed to immense stress from intense emotional engagements with dying patients and bereaved families [1]. The constant exposure to death, grief, and loss can result in burnout [2, 3], “a state of exhaustion in which one is cynical about the value of one’s occupation and doubtful of one’s capacity to perform” [4]. Burnout combined with the vicarious trauma experienced by professional EoL caregivers through the deaths of their patients can further result in compassion fatigue, “an extreme state of tension and preoccupation with the suffering of those being helped to the degree that it can create a secondary traumatic stress for the helper” [5]. If not properly managed, burnout and compassion fatigue can be detrimental to the physical and mental health of professional EoL caregivers, and effects can trickle down to patients, colleagues, family, and friends and pose threats to quality of patient care and quality of personal life.

A recent study found that about 9% of the 7905 surgeons surveyed in the US reported having made a major medical error in the previous 3 months [6] and that stress and burnout were the principal culprits of safety-related quality-of-care concerns [7]. Another study reported that, owing to stress and burnout, the turnover rate of in-patient hospice workers stands at an alarming 30% and reaches as high as 60% for homecare workers [8]. Professional EoL caregiving, by its very nature, necessitates high levels of psycho-socio-emotional competence and therefore adequate support to professional EoL caregivers would entail clinical supervisions or interventions that aim to enhance their sense of autonomy and empathic capacity while providing avenues to cultivate resilience, foster coherence, and achieve meaning-making [9]. Of particular importance is establishing a communal platform for EoL care professionals to periodically reflect on their own attitudes, feelings, and anxieties related to death and loss [10] while being able to express their thoughts to team members for building mutual respect, understanding, and communal support [11].

### Clinical supervision and art therapy

Although supervision has been shown to be effective in reducing fatigue among hospice workers [12], specific attention to emotion-focused coping skills has proven more effective than problem-focused strategy skills in reducing burnout [13]. Emotion-focused supervision requires professionals to consider and communicate feelings and experiences that may be difficult to verbalize. As art provides the means to express oneself through images and metaphors that transcend the barriers of language [14], emotion-focused supervision that incorporates expressive art techniques for self-reflection and self-expression can promote and enhance the understanding of one’s emotions and stress [15]. In other words, supervised art therapy allows art-making and creativity to take central roles in professional self-understanding [16]. Several art therapists have documented how their work with EoL care professionals prevented and reduced burnout by managing stress, fostering collegial connections, emphasizing self-care, and enabling the expression of grief [17, 18].

To better support professional EoL caregivers with work-related stress in Asia, our research team had previously designed a novel art therapy–based supervision model that aimed at alleviating burnout, nurturing emotional awareness, increasing collegial connections, and allowing a safe space to reflect on death and loss [19]. The efficacy of this model was tested through a quasi-experimental design (*n* = 132); 69 participants were enrolled in an 18-h art therapy–based supervision group (run in 6 consecutive weekly sessions lasting 3-h each) and 63 participants were enrolled in an 18-h standard skills–based supervision group (run in 3 consecuitve daily sessions lasting 6-h each). Pre- and post-intervention assessments of participants’ levels of burnout, emotional regulation, and death attitudes revealed significant reductions in exhaustion and death anxiety as well as significant increases in emotional awareness for those enrolled in the art therapy supervision group [20]. This study provided strong evidence that art therapy–based supervision is effective in enhancing emotional regulation, fostering meaning-making, and promoting self-reflection [21].

### Mindfulness practice and art therapy–based supervision

In the past decade, both researchers and clinicians have become increasingly interested in mindfulness practice as studies continue to reveal its beneficial effects [22]. Jon Kabat-Zinn, the foremost pioneer in the therapeutic application of mindfulness, defines it as “the awareness that emerges through paying attention on purpose, in the present moment, and nonjudgmentally to the unfolding of experience moment to moment” [23]. Bishop et al. elaborated that mindfulness is “characterized by curiosity, openness, and acceptance” [24]. Essentially, mindfulness requires individuals to practice a concentrated focus on the present moment, to immerse oneself in his or her immediate experience, and to be freed of the destructive thoughts of future worries and past regrets, so as to attain greater clarity, self-acceptance, and ultimately an enhanced compassion for self and others. As such, the practice of mindfulness can attune seamlessly with the emotional and spiritual needs of those who work with death and loss on a consistent basis.

The literature has provided a robust pool of evidence supporting the efficacy of mindfulness practice and self-compassion in promoting psychological well-being [25], reducing depression and anxiety [26], and enhancing health and overall physical functioning [27]. Despite these positive findings, relatively little research has attempted to integrate mindfulness practice with art therapy to investigate their combined effects for health elevation and stress reduction, an endeavor that warrants much greater attention [28]. The introspective and intuitive foundation of mindfulness practice can complement and facilitate the expressive and creative foundation of art therapy, and vice versa. A unique amalgamation of these two modalities in the context of supportive intervention for EoL care professionals has immense potential to aid them in coping with and rising above the trauma of loss and grief that they face on a daily basis.

In essence, the integration of art therapy and mindfulness practice for addressing the self-care needs of professional EoL caregivers is a scarcely explored area in the field of palliative, hospice, and bereavement care. Building on the clinical foundation of art therapy–based supervision [19–21], a novel mindful-compassion art therapy (MCAT) for clinical supervision was developed. MCAT is a highly structured, multimodal, group-based intervention that aims at creating a supportive platform for EoL care professionals to deeply reflect on and creatively express their experiences of stress and self-care, competence and challenges of caregiving, and the emotionality of loss and grief. It is hypothesized that these interactive processes will serve to foster self-understanding, connectedness, internal strength, and compassion among participants in MCAT, so as to derive a renewed appreciation of the meaning of work as well as enhanced quality of life. The ultimate goal of MCAT is to offer a viable supported self-care intervention to alleviate burnout and cultivate sustained resilience for EoL care professionals in the global context.

## Aim and objectives

Based on the Medical Research Council guidance for developing and evaluating complex intervention [29], the aims of the present study are to assess the feasibility, acceptability, and potential effectiveness of MCAT in reducing burnout and promoting resilience and holistic wellness among a purposive sample of EoL care professionals in Singapore. The specific objectives are the following:

- to assess the effectiveness of MCAT for reducing burnout and negative death attitudes among EoL care professionals, including hospice and palliative care physicians, nurses, social workers, and allied health workers;
- to assess the effectiveness of MCAT for increasing EoL care professionals’ self-perceived levels of emotional regulation, resilience, compassion, and quality of life;
- to assess the feasibility and acceptability of MCAT as a supportive self-care program for EoL care professionals in the context of Singapore; and
- to develop a standardized protocol for further empirical research that tests intervention effectiveness, feasibility, and acceptability of MCAT in other international contexts.

## Methods

### Study design

**Fig. 1:**
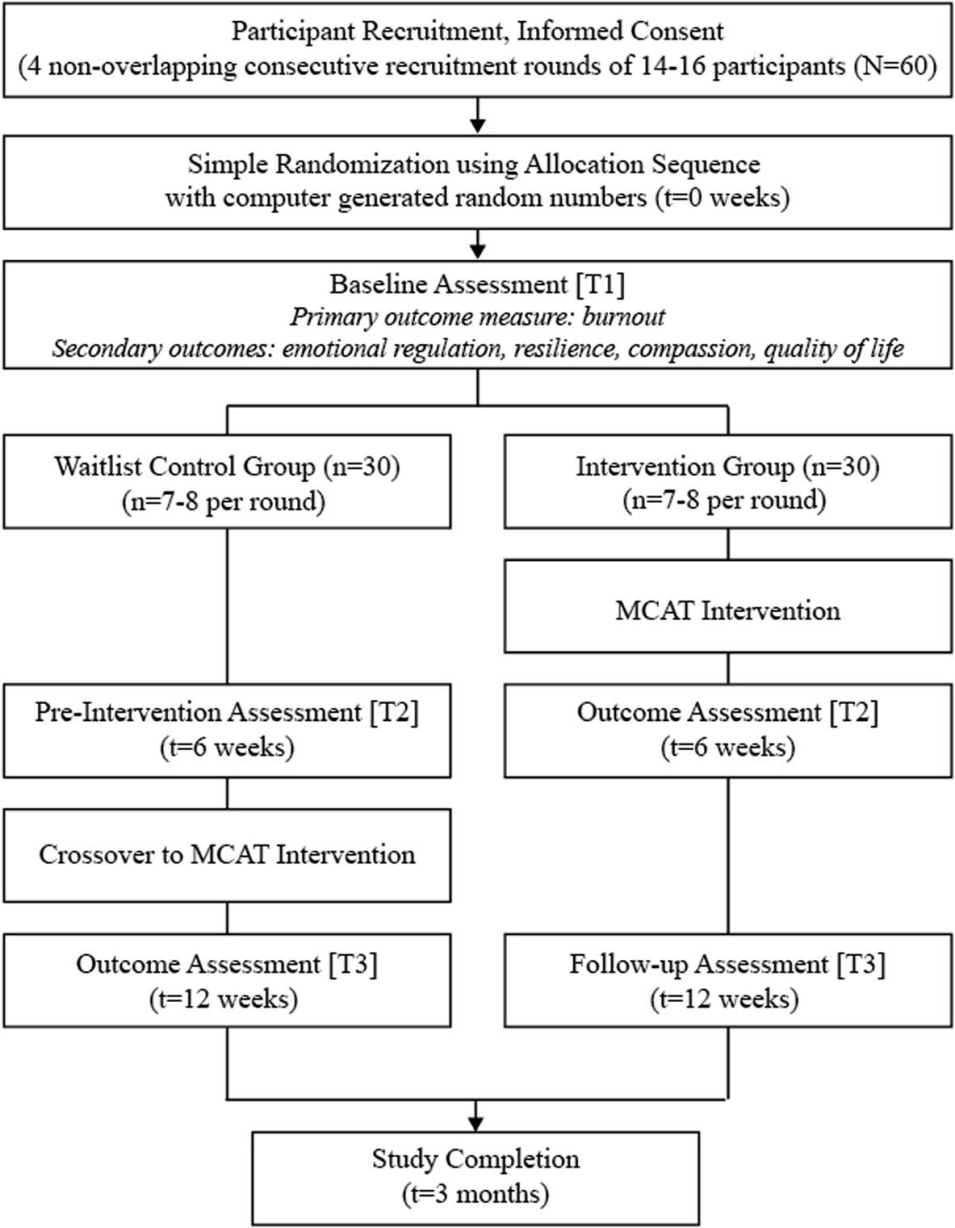
Study design flowchart

This study will adopt an open-label waitlist randomized control trial (RCT) design comprising two groups: (i) an intervention group (which receives MCAT immediately after baseline assessment) and (ii) a waitlist-control group (which receives MCAT immediately after the intervention group completes treatment). A waitlist RCT is chosen on the basis of the ethical principle of justice as the research team hypothesizes and anticipates MCAT to be an effective intervention for reducing burnout and promoting wellness on the basis of previous research on the subject [19–21]. A SPIRIT checklist of the RCT is provided in the Additional file 1. Consenting participants to be recruited from one study site will be randomly allocated to one of these two groups after baseline assessments have been conducted. Figure 1 details the recruitment, assessment, intervention, and follow-up procedures.

### Study site

Study participants will be recruited from the largest homecare hospice service provider and its various satellite centers across Singapore. Specifically, HCA Hospice Care (HCA) (formally known as the Hospice Care Association) is a registered charity that offers homecare and daycare hospice services to terminally ill patients and their families. With one major daycare center (which also serves as the headquarters) and four satellite service centers stationed across the country, they provide nationwide coverage to all Singapore residents who have a diagnosed life-limiting illness. Apart from offering medical care to patients of all ages, HCA provides psychological support and bereavement support services for patients and their families. HCA employs a team of about 60 palliative care specialists, including physicians, nurses, social workers, counselors, allied health professionals, personal care workers, and program staff, to provide round-the-clock support to individuals and families facing the end of life.

### Participants

The sample will comprise 60 EoL care professionals. Recruitment will be conducted at all five HCA service centers (i.e., one daycare and four satellite service centers) concurrently via four non-overlapping consecutive recruitment rounds. Each round will recruit 14 or 16 participants; seven or eight participants will be randomly assigned to one intervention group, and seven or eight participants will be randomly assigned to one control group, adhering to a waitlist RCT design. Participants will be recruited through the medical director of HCA and his appointed staff with the support of a study initiation presentation conducted by the research team during a regular monthly organization meeting with all clinical and program staff. This presentation serves as a study invitation, informing potential participants of MCAT’s basic background and essential research procedures, and includes the request for participants to keep their MCAT experiences private until study completion so as to reduce the potential for intervention contamination. Participants will be invited to join the research with the understanding that they will be given allocated time during regular working hours to participate in the MCAT intervention without any financial implications on their part; they are also assured that refusal to participate is respected and would not result in any negative consequences. Inclusion criteria include EoL care professionals (at least 21 years old) who are fluent in written and spoken English. Exclusion criteria include the inability to provide informed consent or major depression (or other mental health conditions) or both. Written informed consent will be obtained from all participants before study participation. Participants’ confidentiality will be safeguarded under this ethical provision.

### Randomization

Simple randomization for each recruitment round will be conducted by using an allocation sequence based on a computer-generated list of random numbers. Specifically, a random number sequence ranging from 1 to 14 or 16 (depending on the number of participants recruited in each recruitment round) will be generated via Research Randomizer [30]. Thereafter, each participant will be randomly assigned a unique number from the sequence. Participants whose numbers occupy the first seven or eight slots in the sequence will be assigned to the intervention group whereas participants whose numbers occupy the last seven or eight slots will be assigned to the waitlist-control group.

### Sample size calculation

Given an attrition rate of 15% at follow-ups, a sample of 60 (*N* = 60) will provide 80% power to detect an effect size of 0.8 (referencing highly effective psychotherapy studies) [31] on the primary outcome measure of the Maslach Burnout Inventory–General Survey (MBI-GS) [4] between the intervention and control groups at a (two-tailed test) 5% level of significance.

### Intervention group

MCAT will be delivered by an accredited art therapist with a master’s degree in art therapy, together with one clinical researcher with a doctorate in thanatology who is also a certified mindfulness practitioner. The two MCAT therapists will work together and collaboratively to render a full 6-weekly, 18-h, group-based intervention. Each MCAT group will be heterogeneous and include a mix of seven or eight participants who may be physicians, nurses, social workers, and allied health and program workers. Each of the 6-weekly MCAT sessions will cover a unique topic that aims to promote understanding, acceptance, and compassion of self and others for cultivating resilience and shared meaning. The topics will be strategically designed to build upon one another week by week with a scaffolding of themed meditations, guided visualizations, and art activities that deepen participants’ cognitive awareness and emotionality, empowering them to fully experience and appreciate the authenticity and immediacy of their self-reflection, creative expression, and group sharing. Table 1 provides a summary of the topics and experiential activities of the 6-week MCAT intervention model.

**Table 1:** Overview of therapeutic elements of the mindful-compassion art therapy intervention model

Session	Topic	Mindfulness meditation	Visualization theme	Art therapy activities
Week 1	Self-care	Affectionate breathing	Self-kindness	Mandala of self-care / reflective art observations
Week 2	Stress management	Compassionate body-scan	Bodily stress	Symbol of stress / transformative art-making
Week 3	Positive patient care	Loving-kindness meditation	Strengths and progress in patient care	Symbol of strength / creative response writing
Week 4	Challenging patient care	Loving-kindness meditation	Weakness and stagnation in patient care	Symbol of weakness / creative response writing
Week 5	Loss and grief	Meditation on impermanence	A patient’s death	Symbol of grief / collective small group mural
Week 6	Professional purpose	Meditation on giving and receiving compassion	Wisdom learnt and meaning of work	Mandala of meaning / collective large group mural

In week 1, MCAT participants will be invited to explore and examine the topic of positive self-care, beginning with a brief introduction to the concepts of mindfulness practice and expressive art for healing, followed an affectionate breathing meditation, a guided visualization on the theme of self-kindness, a facilitated art-making session for creating a mandala of self-care, and ending with reflective art observation with group sharing. A mandala, defined as a sacred circle across many spiritual traditions, is chosen as for the first art activity because the contained circular space serves as a creative constraint to promote greater creativity and self-expression among participants.  Week 2 will involve an exploration of the topic of stress management, supported by a compassionate body-scan meditation, a guided visualization on the theme of bodily stress, followed by a facilitated art-making session for creating a symbol of stress, and ending with a transformative art-making session on easing stress with group sharing. Weeks 3 and 4 will progress deeper into participants’ cognitive awareness to focus on the topics of patient-care experiences and skill building. Both weeks will begin with a loving-kindness meditation, followed by a guided visualization on a positive patient-care interaction and a facilitated art-making session on the theme of strengths and progress in week 3, and a guided visualization on the theme of challenging patient-care interaction and a facilitated art-making session on the theme of weaknesses and stagnation in week 4, and both weeks will end in creative response writing activities and group sharing. In week 5, participants will be empowered to dive deeper into their emotionality by exploring the topic of loss and grief, beginning with a meditation on impermanence, a guided visualization on the theme of a patient’s death, followed by a facilitated art-making session for creating a symbol of grief, and ending with a collective small group mural activity and group sharing. In week 6, participants will be invited to consolidate all of their experiences and learnings for weeks 1 to 5 and apply them in exploring the topic of professional purpose. This will begin with a meditation on giving and receiving compassion, a guided visualization on the theme of wisdom learnt and meaning of work, followed by a facilitated art-making session for creating a mandala of meaning, and ending with a collective group mural activity and group sharing.

In terms of the specific intervention procedures of MCAT, each weekly session will last 3 h and will comprise an integrative alignment of experiential activities that aim to foster self-reflection, creative expression, authentic sharing, and perspective widening. Table 2 provides a summary of the intervention procedures of a 3-h MCAT session, which is conducted in a medium-size activity room at the research site, equipped with moveable chairs, tables, and a collection of art materials, including acrylic paints, water color, oil and soft pastels, color pencils and markers, crayons, polymer clay, and collage materials such as old magazines, cutouts, scissors, glue, and drawing blocks. These art media serve to promote active engagement among participants of varying levels of experience and skill with the arts.

**Table 2:** Mindful-compassion art therapy weekly session plan

Mindful-compassion art therapy activity	Time allocation	Interventionist
Check-in and review (MP and AT)	10 min	AT and MP
Mindfulness meditation with guided visualization (MP)	20 min	MP
Facilitated art-making (AT)	50 min	AT
Break	15 min	-
Mindful breathing	5 min	MP
Reflective art observation, transformative art, creative Response writing, collective mural	30 min	AT
Small group discussion	20 min	AT and MP
Large group discussion	25 min	AT and MP
Mindful breathing and checkout	5 min	MP

*Abbreviations*: *AT* art therapist, *MP* mindfulness practitioner

Each MCAT session will begin with a 10-min check-in that includes a brief introduction to the topic and activities that are covered for the day. Thereafter, the mindfulness practitioner will lead a 20-min theme-based mindfulness meditation with a guided visualization activity on specific topics as described in the preceding section and this will be immediately followed by a 50-minutue individual art-making session facilitated by the art therapist. During this time, participants will be reminded that beauty in the MCAT therapeutic space is defined by the ability to connect with and freely express their inner emotions via the creation of a tangible art object and thus the focus is on relational aesthetics rather than visual aesthetics [32]. At the 70-min mark of the 3-h session, participants will be reminded of the time and asked to put the final touches on their art pieces in a way that they are comfortable with. After a 15-min break followed by a brief 5-min mindful-breathing exercise, participants will be invited to engage in a 30-min creative activity which may involve (a) reflecting on their own art while observing the arts of others to develop an appreciation of emotional expressions (i.e., reflective art observation), (b) transforming the art objects that they have created before the break into a new art piece (i.e., transformative art), (c) observing and responding to each art object created by all of their peers with one or two written emotive words – using the response words provided by their peers, participants write a short poem or prose to describe their own art piece (i.e., creative response writing), or (d) breaking into small groups of two or three participants who created similar art objects in terms of color, shape, and themes to create a group mural elaborated by a piece of creative writing composed jointly by all group members (i.e., collective mural). Afterwards, there will be a 20-min small group discussion followed by a 25-min large group discussion, where participants are provided with the opportunity to openly share, discuss, and reflect on their creative experiences as well as the stories behind their art. The 3-h session will end with a 5-min mindful-breathing activity with checkout. If a participant expresses a desire to withdraw from the study because of emotional distress at any time during the intervention, the research team will respect their decision and provide a referral to support services rendered by the research site.

### Waitlist-control group

Participants in the waitlist-control group will receive the full 6-week MCAT intervention upon completion of all treatment procedures by intervention group and after the second baseline assessment.

### Procedures

The HCA recruitment team will be asked to distribute research information pamphlets to all eligible staff at the beginning of each recruitment round. Those who are interested to join the study will inform the HCA team and provide their verbal consent in releasing their contact information to the clinical research team. Once they recruit a sufficient number of participants for each recruitment cycle, the HCA team will forward a consolidated information sheet containing only participants’ names and telephone numbers to the clinical research team. An appointed member of the clinical research team will contact the participants and arrange a time to obtain written informed consent and conduct a face-to-face self-administrated baseline assessment (T1) followed by randomization and confirmation of the intervention schedule. Those assigned to the intervention group will attend 6-weekly MCAT sessions within 1 week after randomization; thereafter, immediate post-intervention assessment will be carried out (T2) and there will be a follow-up assessment at 6 weeks (T3). Those assigned to the waitlist-control group will complete a pre-intervention assessment at 6 weeks or after the intervention group completes all treatment procedures (T2) and thereafter attend the 6-weekly MCAT sessions followed by an immediate post-intervention assessment (T3). All MCAT interventions are conducted in the same activity room at the headquarters of HCA. Study participants' personal information will be kept confidential and only the corresponding researchers will have access to such information.

### Outcome measures

**Fig. 2:**
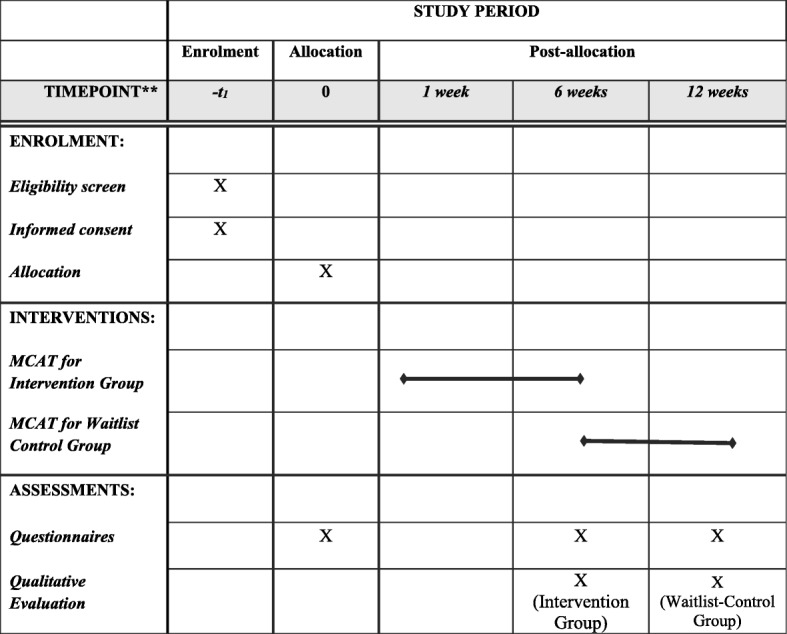
Schedule of enrollment, intervention, and assessment

The primary outcome measure will be participants’ reported level of burnout assessed by the MBI-GS [4]. The MBI-GS comprises 16 items clustered in three subscales: (a) emotional exhaustion, which describes feelings of being physically and emotionally depleted; (b) cynicism, which describes an indifferent attitude and feelings of skepticism toward the nature and significance of one’s work; and (c) professional efficacy, which describes feelings of achievement and confidence in one’s work. The MBI-GS has been shown to have strong validity and reliability [33]. Secondary outcomes will be assessed by using the Death Attitude Profile-Revised [34] (death attitude), the Five Facet Mindfulness Questionnaire [35] (emotional regulation), the Ego-Resilience Scale [36] (resilience), the Self-Compassion Scale Short-Form [37] (compassion), and the World Health Organization Quality of Life Scale-8 [38] (quality of life). See Fig. 2 for schedule of enrollment, intervention, and assessment.

### Demographic measures

To determine whether demographic variables are potential confounding factors that affect primary and secondary outcome variables, basic demographic data (i.e., age, gender, ethnicity, marital status, education, and religion) will be collected from participants during baseline assessment. Information regarding nature of employment (i.e., full-time and part-time), professional roles (i.e., physician, nurse, social worker, personal care worker, psychological staff, and program staff), and years of professional experience in EoL care will also be collected at baseline.

### Qualitative outcome evaluation

Together with the quantitative assessment, a series of simple open-ended evaluative questions will be used to seek participants’ insights into their experience with the MCAT intervention, the impact of MCAT on their professional and personal lives, and their views on taking part in this study. Participants will be asked to provide written responses to these questions during the immediate post-intervention assessment. Also, large group sharing from each MCAT intervention session and all reflective writings produced during the intervention will be recorded, documented, and transcribed for analysis to identify insights into concerns which might impact intervention effectiveness. One in two group sharing transcripts will be randomly selected for review by the principal investigator and one co-investigator for data monitoring and quality and safety assurance.

### Acceptability and feasibility assessment

To assess feasibility in implementation and delivery of MCAT in community settings, the following will be recorded: (a) time needed to organize and conduct the intervention sessions and transcribe group sharing, (b) deviations from the intervention protocol, (c) uncompleted interventions and their reasons, and (d) MCAT interventionists’ perceptions of competence. MCAT interventionists will also record the fidelity of the intervention, experiences of intervention delivery, observations of participants’ experiences and responses during and after intervention, and any difficult or deviant cases. To assess acceptability, two acceptability focus groups comprising six to eight randomly selected MCAT participants will be conducted after completion of all interventions to elicit (1) experiences of intervention, (2) insights into which intervention components work best for them and under what circumstances, (3) insights into concerns which might impact intervention effectiveness, (4) factors that draw their participation and sustained engagement, (5) factors that deter them from sustainable practice, (6) factors that may lead other EoL care professionals to be more inclined to partake in such a program, and (7) ideas and suggestions to make the program more appealing to different types of care workers. All focus groups will be recorded and transcribed verbatim for analyses. Finally, data generated from the qualitative evaluation will serve to inform the acceptability and feasibility study.

### Data analysis

#### Quantitative data

All quantitative data will be entered, stored, and analyzed by using SPSS statistical analysis software. Between- and within-subject comparisons of outcomes will be conducted by using repeated-measures analysis of variance (ANOVA) with the appropriate confidence interval and effect size estimates reported. Intervention and waitlist-control groups will be compared to assess intervention effectiveness on the primary outcome of burnout (continuous variable); secondary outcomes, including death attitude, emotional regulation, resilience, compassion, and quality of life (continuous variables), will also be compared to examine the potential effects of MCAT on other domains of psycho-socio-spiritual well-being. Between-subject comparisons will be conducted at T2 with baseline assessment. To explore the potential maintenance of intervention effects, within-subject comparisons will be conducted with the intervention group at T3 with baseline assessment. To examine the overall intervention effects of MCAT, within-subject comparisons will be conducted by combining the baseline-to-T2 assessment scores for the intervention group and the T2-to-T3 assessment scores for the waitlist-control group. Multi-level analysis will be conducted to characterize and predict changes in all outcomes. Intervention and waitlist-control groups will also be compared on demographic characteristics and baseline measures. If necessary, these will be controlled in the analyses. Exploratory analyses on the influence of nature or work and years of professional EoL care experience on main outcomes and secondary outcomes will also be carried out. Finally, recruitment rates as well as comparisons of dropout rates and missing data in the two groups will be reported.

#### Qualitative data

The framework method of analysis will be used [39]. Analysis is both deductive (from pre-set aims and objectives) and inductive (arising from participants’ views). This method tends to be more structured than some other methods of qualitative analysis, and the process is more explicit and more informed by *a priori* questions. It is designed so that it can be easily understood and assessed by people other than the analysts, such as funding bodies, policy makers, and participants. Throughout the analytical process, strategies to maximize credibility, criticality, and authenticity are applied. The QSR NVIVO software package will be used to manage the data.

## Discussion

This study has been approved by the institutional review board of Nanyang Technological University Singapore (IRB-2015-04-021) and is currently in the data collection phase. The aim of this study is to test the effectiveness of a novel, evidence-based, multimodal psycho-socio-spiritual intervention for supporting EoL care professionals who are consistently faced with the death and loss of their patients. This is also the first known study that integrates mindfulness meditation with art therapy to form a unique and highly structured intervention with clear and meticulous delivery procedures that address the urgent problem of professional burnout and compassionate fatigue in the fields of EoL, palliative, and bereavement care while promoting resilience, meaning in work, and quality of life to promote sustained employment and reduce turnover rates. It addresses a critical gap in the self-care and supportive care literature for professional caregivers. Despite its merits, it has a number of limitations. First, the sample of this RCT is relatively small because of resource constraints, and sample size calculation is based on a large effect size referencing highly effective psychotherapy studies [31]. Second, this study recruits trained medical and social care professionals only and thus intervention appropriateness for family caregivers and volunteers, who represent a major stakeholder group in EoL care, is unknown. Third, this is a single-site study and its applicability and transferability to other care settings such as acute hospitals and in-patient hospices remain unclear. To address these limitations, future studies need to expand the scale and scope of research to include a larger sample size of all EoL care stakeholders by using a multi-site design. Nonetheless, the outcomes of this study will contribute to advancements in both theories and practices for supporting professional EoL care professionals around the world. It will also inform policy makers about the feasibility and acceptability of delivering such an intervention in real-world community-based institutional settings, which will enable future studies to carry out long-term benefit and cost-effectiveness analysis.

## Trial status

As of this writing, the RCT is in month 40 of 48 planned months of research and data collection.

## Additional file

Additional file 1: SPIRIT (Standard Protocol Items: Recommendations for Interventional Trials) Checklist. (DOC 125 kb)

## Data Availability

Not applicable.

## Abbreviations

EoL: End-of-life
HCA: HCA Hospice Care
MBI-GS: Maslach Burnout Inventory–General Survey
MCAT: Mindful-compassion art therapy
RCT: Randomized control trial
T: Time point
